# Non-inferiority of dose reduction versus standard dosing of TNF-inhibitors in axial spondyloarthritis

**DOI:** 10.1186/s13075-018-1772-z

**Published:** 2019-01-08

**Authors:** Jordi Gratacós, Caridad Pontes, Xavier Juanola, Jesús Sanz, Ferran Torres, Cristina Avendaño, Antoni Vallano, Gonzalo Calvo, Eugenio de Miguel, Raimon Sanmartí, The REDES-TNF investigators, Miriam Almirall, Maria Aparicio, Agustí Sellas, Roser Vives, Nestor Albiñana, Mireia Moreno, Teresa Clavaguera, Juan Carlos Torre-Alonso, Raúl Veroz, Carlos Rodríguez-Lozano, Luís Francisco Linares, Ana Urruticoechea, Eduardo Collantes, Rosa María Morlà, Dèlia Reina, Eduardo Cuende, Pedro Zarco, Maria Cruz Fernández-Espartero, Rosario García-Vicuña, Carlos Alberto Montilla, Alejandro Villalba, Dora Pascual, Cristina Campos, Antonio Juan, Rafael Ariza, Consuelo Díaz-Miguel, Manuel Maqueda, Maria Pilar Fernández-Dapica, Manuel Fernández-Prada, Enrique Batlle, Carlos González-Fernández, Rubén Queiro

**Affiliations:** 1 Rheumatology Department, Hospital de Sabadell, Institut Universitari Parc Taulí, Universitat Autònoma de Barcelona, Sabadell, Barcelona Spain; 2 Clinical Pharmacology Unit, Hospital de Sabadell, Institut Universitari Parc Taulí, Universitat Autònoma de Barcelona, c/Taulí n°1, 08208 Sabadell, Barcelona Spain; 3 Rheumatology Department, Hospital Universitario de Bellvitge, Universitat de Barcelona, Bellvitge, Barcelona Spain; 4 Rheumatology Department, Hospital Universitario Puerta de Hierro- Majadahonda, Madrid, Spain; 5 Medical Statistics core facility, IDIBAPS, Hospital Clínic, Biostatistics Unit, School of Medicine, Universitat Autònoma de Barcelona, Barcelona, Spain; 6 Clinical Pharmacology Department, Hospital Puerta de Hierro Majadahonda, Madrid, Spain; 7 Clinical Pharmacology Department, Hospital Universitario de Bellvitge - Universitat de Barcelona, Barcelona, Spain; 8 Clinical Pharmacology Department, Hospital Clínic de Barcelona - Universitat de Barcelona, Barcelona, Spain; 9 Rheumatology Department, Hospital Universitario La Paz, Madrid, Spain; 10 Rheumatology Department, Hospital Clínic de Barcelona - Universitat de Barcelona, Barcelona, Spain

**Keywords:** Spondyloarthritis, Dose-tapering, Non-inferiority, TNF inhibitors

## Abstract

**Objective:**

The objective was to determine if dose reduction is non-inferior to full-dose TNFi to maintain low disease activity (LDA) in patients already in remission with TNFi, in axial spondyloarthritis.

**Methods:**

Randomized, parallel, non-inferiority, open-label multicentre clinical trial. Patients were eligible if they had axial spondyloarthritis and had been in clinical remission for ≥ 6 months with any available TNFi (adalimumab, etanercept, infliximab, golimumab) at the dose recommended by product labelling. Patients were randomized by automated central allocation to continue the same TNFi dose schedule, or to reduce the dose by roughly half according to the protocol. The main outcome was the proportion of subjects with LDA after 1 year. Serious adverse reactions or infections were recorded.

**Results:**

The trial stopped due to end of the funding period, after 126 patients were randomized; 113 patients (84.1% male, mean age (SD) 45.6 (13.0) years) were included in the main per-protocol subset. Non-inferiority was concluded for LDA at 1 year (47/55 (83.8%) patients in the full-dose and 48/58 (81.3%) patients in the reduced-dose arm, adjusted difference (95% CI) − 2.5% (− 16.6% to 11.7%)). Serious adverse reactions or infections were reported in 7/62 patients (11.3%) assigned to full dose and 2/61 patients (3.3%) assigned to reduced dose (*p* value = 0.164).

**Conclusion:**

In patients with ankylosing spondylitis in clinical remission for at least 6 months, dose reduction is non-inferior to full TNF inhibitor doses to maintain LDA after 1 year. Serious adverse events may be less frequent with reduced doses.

**Trial registration:**

EU Clinical Trials Registry, EudraCT 2011–005871-18 and ClinicalTrials.gov, NCT01604629.

**Electronic supplementary material:**

The online version of this article (10.1186/s13075-018-1772-z) contains supplementary material, which is available to authorized users.

## Introduction

Spondyloarthritis (SpA), a group of rheumatic diseases that share immunogenic, clinical and radiological characteristics, are classified as axial (axSpA) or peripheral by the Assessment of Spondyloarthritis International Society (ASAS). Ankylosing spondylitis (AS) is the main disease within SpA, with a prevalence of 0.4–0.9% in Caucasians, and marked differences by race, prevalence of HLA B27, and geographical area [1, 2]. Despite treatment, many Patients with SpA have impaired quality of life and work disability [3, 4]. SpA management aims to achieve and maintain clinical remission or low disease activity (LDA) [5]. The first step is non-steroidal anti-inflammatory drugs (NSAIDs) and physical therapy, although 60% of patients obtain no clinical benefit [6]. Disease-modifying anti-rheumatic drugs (DMARDs) are not deemed efficacious, but tumour necrosis factor inhibitors (TNFi) can induce and sustain long-term clinical remission in patients not responding to NSAIDs [7]. However, the optimal duration and dose for long-term treatment remain unclear, and the recommended TNFi doses are the same for treatment of acute and chronic disease. Although the risk-benefit is favourable, prolonged treatment with TNFi chronically suppresses the immune response, increasing the risk of serious infection dose-dependently, especially in frail patients. It is unclear whether chronic use may increase malignancies and neurological demyelinating diseases [8–10]. Finally, TNFi are expensive and a cost burden.

Discontinuation of TNFi after achieving clinical remission is followed by early relapse in most cases [11–13]. However, lower doses of TNFi may be sufficient in patients with LDA and low levels of inflammatory mediators, according to uncontrolled or observational studies [14, 15]. Some clinical guidelines recommend empirical dose reductions despite a lack of robust supportive evidence [16]. Likewise, although drug-level monitoring may help physicians optimize and prevent overtreatment in patients with rheumatoid arthritis treated with TNFi [17], there are no conclusive data on useful biomarkers for the monitoring of TNFi in AS [18].

The objective of this study was to determine the suitability of dose reduction for long-term treatment with TNFi in axSpA and potential predictors of poor clinical response. The main study hypothesis was that in patients with axSpA in persistent clinical remission with TNFi, reduced TNFi doses would be not inferior to full doses, as assessed by the proportion of patients not reaching LDA criteria for changing treatments. In addition, we analysed pre and post withdrawal drug levels, and markers of inflammation and bone markers in a subset.

## Patients and methods

A prospective multicentre, parallel, controlled and randomized open-label clinical trial was conducted at 22 Spanish centres. Patients were eligible if they met criteria for axSpA according to the ASAS classification criteria [19], on treatment with the recommended doses of commercially available TNFi (infliximab, adalimumab, etanercept or golimumab) at the time of the study, and in sustained clinical remission defined as Bath Ankylosing Spondylitis Disease Activity Index (BASDAI) ≤ 2, no clinically active arthritis or enthesitis and C-reactive protein (CRP) equal to or higher than the upper limit of normality for ≥ 6 months. Exclusion criteria were secondary SpA or predominantly peripheral arthritis, comorbidity interfering with the clinical assessment, pregnancy and breast feeding. Before any study-related procedure, patients were given comprehensive information on the study objectives, procedures and risks, and written informed consent was obtained from all patients.

### Randomization and treatments

Eligible patients were randomized to receive either the full recommended TNFi dose or a reduced dose according to an agreed protocol supported by clinical practice (Table 1). Patients were screened, and information on previous TNFi treatment, clinical activity and other eligibility criteria entered in the electronic case report form (eCRF). Patients were automatically randomized by central allocation according to a list generated using SAS PROC PLAN v9.2 (SAS Institute Inc., Cary, NC, USA) with a 1:1 assignment ratio between arms, stratifying by prior TNFi treatment in blocks of four elements. The randomization list was loaded to a separate module of the eCRF so that the module automatically assigned the lowest sequential number available within the randomization stratum. An auditable registry of the date, time and other variables related to stratification and treatment assignment was kept. Patients and the study investigators were not blinded to treatment once assigned.

**Table 1:** Study treatments

Anti-TNF drug	Posology according to SPC	Route	Full-dose group	Reduced-dose group
Adalimumab	40 mg every 2 weeks	SC	40 mg every 2 weeks	40 mg every 3 weeks
Etanercept	25–50 mg every 3–7 days	SC	25 mg every 3 days or 50 mg every 7 days	50 mg every 10 days
Golimumab	50 mg every month	SC	50 mg every 4 weeks	50 mg every 6 weeks
Infliximab	5 mg/Kg every 6–8 weeks	IV	5 mg/Kg every 6–8 weeks	3 mg/Kg every 8 weeks

*SPC* Summary of Product Characteristics, *SC* subcutaneous, *IV* intravenous

### Clinical outcomes

The main clinical endpoint was the percentage of patients with LDA (BASDAI score < 4, plus physician global assessment < 4, patient global assessment < 4 and nocturnal axial pain < 4 as assessed on a 0–10 visual analogue scale (VAS)) at one year. The key secondary endpoint was the proportion of patients who remained in clinical remission (BASDAI <= 2, physician global assessment <= 2, and patient global assessment <= 2 with serum CRP below the upper limit of normality) at 1 year. Other secondary objectives were the usual axSpA clinical outcomes (Ankylosing Spondylitis Disease Activity Score (ASDAS)-CRP, ASAS response criteria, BASDAI, functional assessment as measured by Bath Anklylosing Spondylitis Functional Index (BASFI) and quality of life as measured by Ankylosing Spondylitis Quality of Life (ASQoL)). Relapses defined according to ASAS group, based on pain, BASDAI and ASDAS-CRP [20] were calculated *a posteriori* on recorded data. Adverse events, serious adverse reactions requiring hospitalization and/or treatment withdrawal, and serious infections requiring systemic antibiotic treatment and/or hospitalization were recorded.

Blood samples for the measurement of TNFi, anti-drug antibodies and inflammatory mediators were collected within the 24 h before TNFi injection in 18 participating centres. The number of samples requested were ≥ 2 per patient, separated by ≥ 90 days when feasible. Samples were frozen and stored at − 80 °C until shipment at the end of the study.

Inflammatory mediators were assayed at Laboratori de Recerca – I3PT (Sabadell, Spain). Plasma levels of TNF alpha, IL-6, sclerostin (SOST) and Dickkopf-related protein 1 (DKK-1) were determined using a Luminex multiplex immunoassay (Merck-Millipore, Darmstadt) (accuracy = 82–97%, inter-assay and intra-assay precision = < 15% and < 10%, respectively); high-sensitivity CRP (hs-CRP) using a Luminex immunoassay (Merck-Millipore, Darmstadt) (accuracy = 93%, inter-assay and intra-assay precision = < 15% and < 10%, respectively); plasma calprotectin levels using Calprolab ELISA test HRP (Calpro, Lysaker) (inter-assay and intra-assay precision < 7% and < 6%, respectively).

Drug and anti-drug antibody (ADA) levels were determined by assay at the Immunology Department – Hospital La Paz (Madrid, Spain). TNFi was measured by commercially available capture ELISA kits, (Promonitor, Progenika, Derio, Vizcaya), with cutoff values established using serum from 150 healthy blood donors and 100 patients with RA who were naïve to biological drugs. The positivity threshold was mean optical density + 6 standard deviations: positive levels were infliximab > 0.035 μg/ml, adalimumab > 0.024 μg/ml, golimumab > 0.022 μg/ml and etanercept > 0.035 μg/ml. ADAs were assayed only in samples with no detectable TNFi, using a home-made, two-site (bridging) ELISA.

### Visit schedule

Visits were scheduled according to routine clinical practice, although recommended intervals between visits were 8–16 weeks [21]; a mandatory visit was required 1 year after randomization for the main assessment. Follow up continued until the end of study, change in biological treatment, withdrawal of informed consent or loss to follow up. NSAID treatment and dose modification was permitted throughout the study and was recorded. The study protocol has been reported in full elsewhere [22].

### Sample size and statistical methods

The estimated proportion of patients at LDA after 1 year of treatment with full-dose TNFi was 87% in patients who were in stable remission for 4 months [23]. A non-inferiority margin of 17% was established, and protection against type I error = 2.5% (unilateral) and against type II error = 20%, being the estimated sample size to test the non-inferiority hypothesis of the reduced-dose strategy with respect to the full-dose strategy of 85 patients per arm [24, 25]. The non-inferiority (delta margin) of 17% was prospectively set based on the consensus on clinical relevance reached by the rheumatologists involved, who decided that a proportion of patients with acceptable control < 70% after 1 year would severely discourage the use of dose reduction [22]. To ensure the reliability of the study, the lower confidence interval (CI) in the full-dose arm had to be > 60% to confirm non-inferiority, in order to ensure that the control treatment had been reasonably effective [22].

A per-protocol set (PPS) for analysis was the pre-defined primary population for this non-inferiority study, following a conservative approach where any potential differences can be maximized. The principal endpoint and the key secondary endpoints were also tested in the full analysis set (FAS) population for sensitivity. The principal and key secondary end-points were assessed by estimating the between-treatment risk differences after 1 year of randomization and checking these against the pre-defined non-inferiority margin (delta (δ)) of 17%. Rates and risk differences were estimated using log-binomial regression including treatment and the factors used to stratify assignment. The effects of baseline clinical and inflammatory parameters on the results were evaluated, and the heterogeneity of the main results checked for stratification factors. A detailed statistical analysis plan was issued and approved on 25^th^ September 2014. The statistical analysis was run in the SAS System (SAS Institute Inc., Cary, NC, USA) v9.2. The detailed statistical methods have been reported elsewhere [22].

## Results

**Fig. 1:**
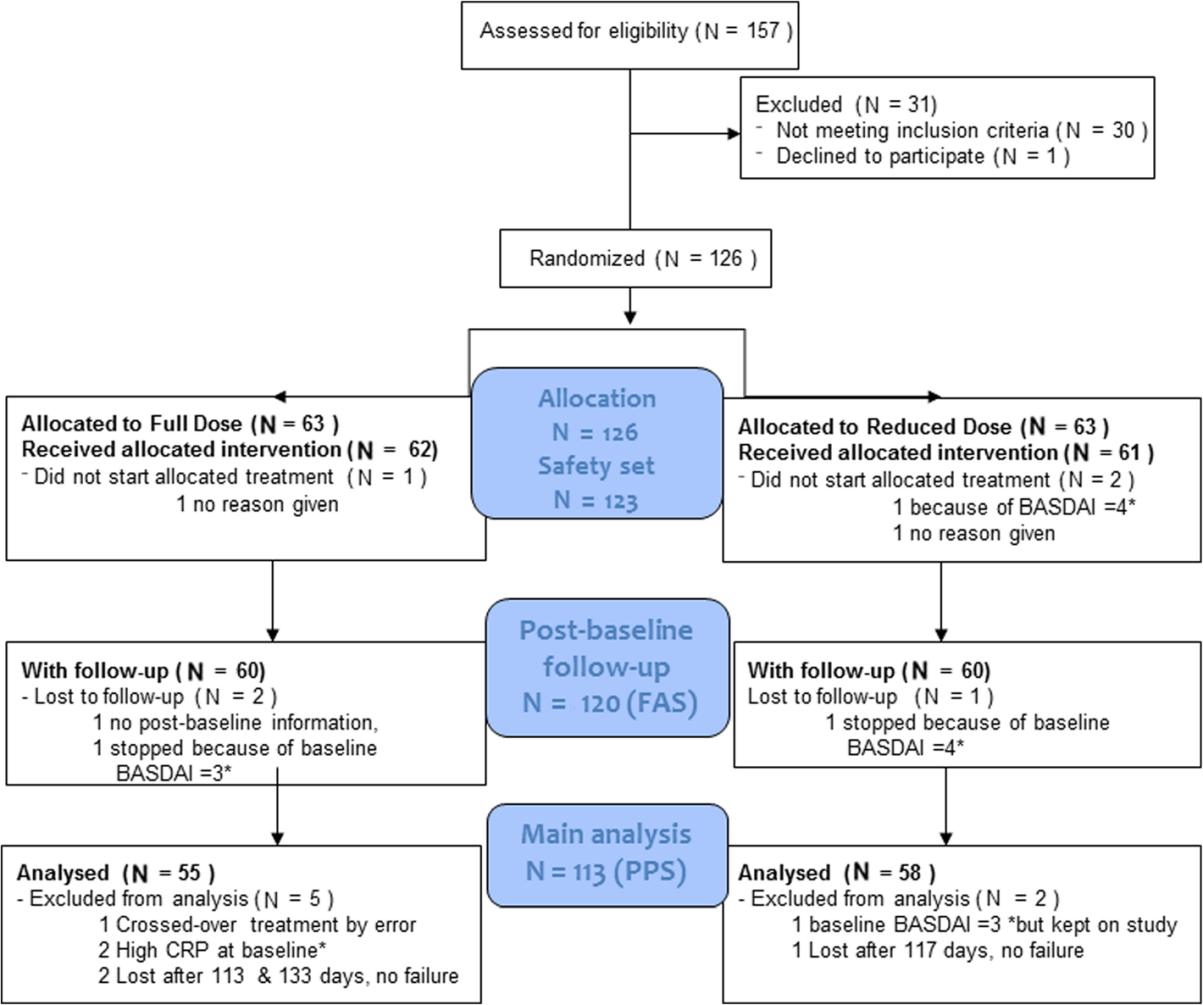
Disposition of patients. FAS, full analysis; PPS, per-protocol main analysis set; BASDAI, Bath Ankylosing Spondylitis Disease Activity Index; CRP, C-reactive protein. *Not meeting inclusion criteria for remission (BASDAI ≤ 2 and CRP ≤ upper limit of normality)

From 6^th^ July 2012 to 15^th^ May 2014, 157 patients were screened. Due to recruitment delays and time restrictions on funding, recruitment was closed after 126 patients had been randomized; the last patient visit was on 10^th^ June 2015. Figure 1 shows the number of patients assigned to each treatment arm (FAS), lost to follow up during the study (total of three patients) and the reasons for exclusion from the PPS main analysis set. Baseline participant characteristics were similar between groups (Table 2). Although the protocol allowed inclusion of patients with axSpA, all included patients fulfilled criteria for AS.

**Table 2:** Baseline characteristics of patients

Baseline characteristics	Full analysis set			Per-protocol set		
	Full dose (*n* = 60)	Dose reduction (*n* = 60)	Total (*n* = 120)	Full dose (*n* = 55)	Dose reduction (*n* = 58)	Total (*n* = 113)
Gender (male), *n* (%)	53 (88.3)	49 (81.7)	102 (85.0)	48 (87.3)	47 (81.0)	95 (84.1)
Age (years), mean (SD)	46.2 (13.7)	43.7 (12.4)	44.9 (13.1)	47.2 (13.6)	43.6 (12.4)	45.6 (13.0)
BMI, mean (SD)	25.9 (3.4)	25.8 (3.8)	25.9 (3.6)	25.8 (3.4)	25.9 (3.8)	25.9 (3.6)
Years from diagnosis, median (P_25_, P_75_)	10.4 (7.1, 20.8)	9.3 (5.2, 17.6)	10.0 (5.9, 19.8)	10.4 (7.1, 22.6)	9.3 (5.0, 19.0)	10.0 (5.9, 20.3)
ASAS criteria for sacroilitis, *n* (%)	58 (96.7)	59 (98.3)	117 (97.5)	53 (96.4)	57 (98.3)	110 (97.3)
ASDAS-CRP, median (P_25_, P_75_)	0.7 (0.5, 1.1)	0.7 (0.5, 1.1)	0.7 (0.5, 1.1)	1.1 (0.8, 3.5)	1.0 (0.7, 1.9)	1.1 (0.7, 2.0)
BASDAI, median (P_25_, P_75_)	1.0 (0.6, 1.7)	1.0 (0.2, 1.4)	1.0 (0.4, 1.6)	1.0 (0.6, 1.7)	1.0 (0.2, 1.4)	1.0 (0.4, 1.6)
VAS nocturnal axial pain, mean (SD)	0.85 (1.0)	1.03 (1.16)	0.94 (1.09)	0.84 (1.01)	1.02 (1.15)	0.93 (1.08)
IGA, median (P_25_, P_75_)	1.0 (0.0, 2.0)	1.0 (0.0, 1.0)	1.0 (0.0, 1.0)	1.0 (0.0, 2.0)	1.0 (0.0, 1.0)	1.0 (0.0, 1.0)
PGA, median (P_25_, P_75_)	1.0 (0.0, 2.0)	1.0 (0.0, 2.0)	1.0 (0.0, 2.0)	1.0 (0.0, 2.0)	1.0 (0.0, 1.0)	1.0 (0.0, 1.0)
Current TNFi, n (%)						
Adalimumab	24 (40.0)	23 (38.3)	47 (39.2)	22 (40.0)	22 (37.9)	44 (38.9)
Etanercept	21 (35.0)	20 (33.3)	41 (34.2)	19 (34.5)	19 (32.8)	38 (33.6)
Golimumab	4 (6.7)	5 (8.3)	9 (7.5)	4 (7.3)	5 (8.6)	9 (8.0)
Infliximab	11 (18.3)	12 (20.0)	23 (19.2)	10 (18.2)	12 (20.7)	22 (19.5)
N of previous TNFi, *n* (%)						
None	50 (83.3)	44 (73.3)	94 (78.3)	46 (83.6)	42 (72.41)	88 (77.9)
One	10 (16.7)	11 (18.3)	21 (17.5)	9 (16.4)	11 (19.0)	20 (17.7)
Two	0 (0.0)	5 (8.3)	5 (4.2)	0 (0.0)	5 (8.6)	5 (4.4)
NSAID use, *n* (%)	16 (26.7)	14 (23.3)	28 (24.8)	15 (27.3)	13 (22.4)	28 (24.8)

*n* number, *SD* standard deviation, *P25* percentile 25, *P75* percentile 75, *BMI* body mass index, *ASAS* Assessment of Spondyloarthritis International Society, *ASDAS-CRP* Ankylosing Spondylitis Disease Activity Score including C-reactive protein, *BASDAI* Bath Ankylosing Spondylitis Disease Activity Index; VAS patient’s rating of nocturnal axial pain by visual analogue scale ranging from 0 (none) to 10 (worst), *PGA* Patient Global Assessment of disease activity rated from 0 (best) to 10 (worst), *IGA* Investigator’s Global Assessment of disease activity rated from 0 (best) to 10 (worst), *TNFi* TNF inhibitor, *NSAID* non-steroidal anti-inflammatory drug

**Fig. 2:**
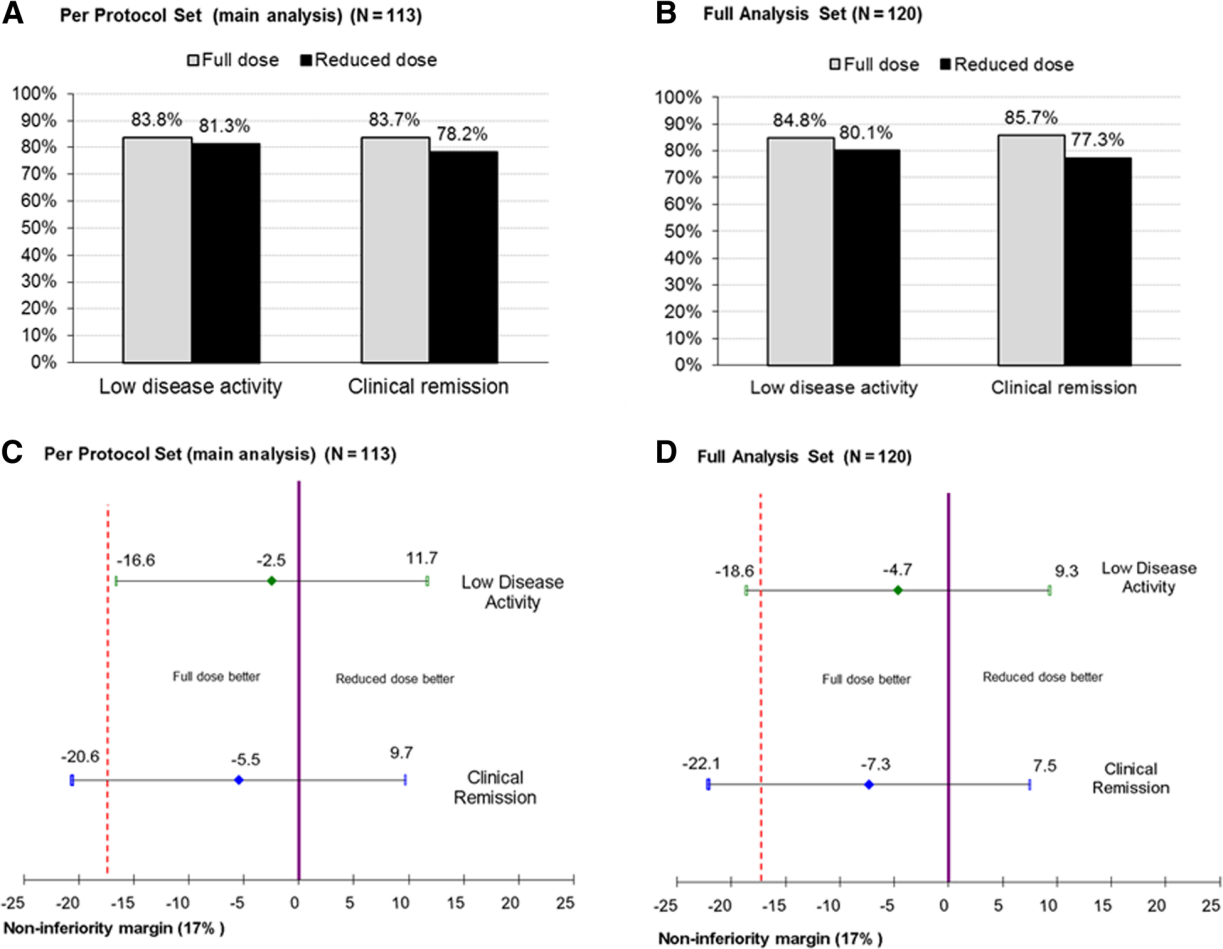
Proportion of patients with low disease activity and clinical remission at 12 months. **a** Proportion of subjects with low disease activity and clinical remission at 12 months, per-protocol subset (main analysis). **b** Proportion of subjects with low disease activity and clinical remission at 12 months, intention-to-treat subset. **c** Adjusted differences between groups and non-inferiority testing for low disease activity and clinical remission, per-protocol subset (main analysis). **d** Adjusted differences between groups and non-inferiority testing for low disease activity and clinical remission, intention-to-treat subset. Low disease activity was defined by Bath Ankylosing Spondylitis Disease Activity Index (BASDAI) < 4 and Physician Global Assessment < 4, Patient Global Assessment < 4 and axial pain at night < 4. Clinical remission was defined by BASDAI ≤ 2, Physician Global Assessment ≤ 2 and Patient Global Assessment ≤ 2

The main study result fulfilled the objective of non-inferiority, with a proportion (95% CI) of patients with LDA at one year of randomization of 83.8% (64.8–102.7%) in the full-dose arm and 81.3% (62.8–99.8%) in the reduced-dose arm, and absolute difference in LDA at 1 year between treatment groups of − 2.5% (95% CI of − 16.6 to 11.7%) in the pre-defined PPS population (Fig. 2a and c). The lower bound of CI for the estimate in the control arm was > 60%, fulfilling the pre-specified validation criteria. The percentage (95% CI) of patients in clinical remission (the key secondary endpoint) at one year of randomization was 83.7% (64.7–102.7%) in the full-dose arm and 78.2% (59.7–96.8%) in the reduced-dose arm, with absolute differences between treatment groups of − 5.5% (− 20.6 to 9.7%) (*p* = 0.480) (Fig. 2a and c). The sensitivity analysis in the FAS population showed similar estimates for the differences between the two arms for the primary and key secondary end-points (Fig. 2b and d). No significant differences were observed between treatment arms for any of the secondary variables (Table 3).

**Table 3:** Secondary endpoints

Efficacy secondary variables at 1 year				
Variables	Full dose (*n* = 55)	Dose reduction (*n* = 58)	Differences between groups	*P* value
ASDAS-CRP < 1.3^a^	61.4% [47.3%; 75.5%]	53.5% [39.7%; 67.3%]	7.8% [−10.0%; 25.8%]	0.389
ASDAS-CRP relapse^a^	6.6% [−5.4%; 18.7%]	12.7% [1.4%; 24.0%]	−6.1% [−25.2%; 12.9%]	0.529
Relapse BASDAI-VAS^a^	15.9% [−3.1%; 34.9%]	10.4% [−9.1%; 29.8%]	5.5% [−12.3%; 23.4%]	0.545
Relapse SER^a^	6.4% [−13.0%; 25.8%]	10.1% [−7.9%; 28.1%]	− 3.7% [− 19.2%; 11.8%]	0.638
NSAIDs use^a^	18.3% [− 1.2%; 37.7%]	20.6% [1.5%; 39.8%]	−2.4% [− 18.9%; 14.2%]	0.779
ASDAS-CRP score^b^	1.1 (0.1) [0.9;1.3]	1.1 (0.1) [0.9;1.3]	0.0 (0.2) [−0.3;0.3]	0.783
BASDAI^b^	1.4 (0.2) [1.1;1.7]	1.4 (0.2) [1.1;1.7]	− 0.0 (0.2) [− 0.5;0.4]	0.890
VAS night axial pain^b^	1.4 (0.2) [1.0;1.8]	1.1 (0.2) [0.7;1.6]	0.3 (0.3) [−0.3;0.9]	0.337
PGA^b^	1.6 (0.2) [1.2;2.0]	1.6 (0.2) [1.1;2.0]	0.0 (0.3) [−0.6;0.6]	0.962
IGA^b^	1.1 (0.2) [0.8;1.4]	1.1 (0.2) [0.8;1.4]	−0.0 (0.2) [− 0.4;0.40]	0.923
BASFI^b^	1.7 (0.2) [1.2;2.1]	1.8 (0.2) [1.3;2.3]	−0.2 (0.3) [− 0.8;0.5]	0.616
ASQoL^b^	2.3 (0.5) [1.2;3.3]	2.2 (0.6) [1.0;3.3]	0.1 (0.7) [−1.3;1.5]	0.858
Safety secondary variables				
Adverse event or infections	Full dose (*n* = 62)	Dose reduction (*n* = 61)	Differences between groups	*P* value
Any^c^	22 (35.5%) [23.6%;47.4%]	17 (27.9%) [16.6%;39.1%]	(7.6%) [−8.8%;24.0%]	0.439
Related^c^	8 (12.9%) [4.6%;21.2%]	5 (8.2%) [1.3%;15.1%]	(4.7%) [−6.1%;15.5%]	0.559
Severe^c^	14 (22.6%) [12.2%;33.0%]	11 (18.0%) [8.4%;27.7%]	(4.6%) [−9.6%;18.7%]	0.655
Severe and related^c^	7 (11.3%) [3.4%;19.2%]	2 (3.3%) [−1.2%;7.7%]	(8%) [− 1.1%;17.1%]	0.164

Ankylosing Spondylitis Disease Activity Score including C-reactive protein (ASDAS-CRP) relapse was defined by increase ≥ 1.1. Bath Ankylosing Spondylitis Disease Activity Index-visual analogue scale (BASDAI-VAS) was defined by increases of 20% or a 2-unit increase in the 0–10 scale. SER relapse was defined by BASDAI ≥ 4, global clinical assessment by physician ≥ 4 and at least one of three following criteria: patient assessment ≥ 4, axial nocturnal pain (VAS) ≥ 4, and increase in acute phase reactants (reactive °C protein (PCR) and/or erythrocyte sedimentation rate (ESR). Ankylosing Spondylitis Disease Activity Score-C reactive protein (ASDAS-C), which is calculated as (0.12 x back pain) + (0.06 x duration of morning stiffness) + (0.11 x patient GA) + (0.07 x peripheral pain/swelling) + (0.58 x Ln(CRP + 1)); if CRP is not available but ESR is available, the last term is changed by (0.29 x √(ESR)). BASDAI is calculated as {A + B + C + D+ [(E + F)/2]}/5 where A to E are 6 VAS, rated 0 (best) to 10 (worst) assessing (A) fatigue, (B) axial skeletal pain, (C) peripheral joint pain, (D) pain on contact or pressure, (E) intensity of morning stiffness and (F) duration of morning stiffness; VAS nocturnal axial pain is the patient’s rating of nocturnal pain by VAS ranging from 0 (none) to 10 (worst). Patient Global Assessment (PGA) of disease activity was rated from 0 (best) to 10 (worst). Investigator’s Global Assessment (IGA) of disease activity was rated from 0 (best) to 10 (worst). The Bath Ankylosing Spondylitis Functional Index (BASFI) is calculated as the average value of answers to 10 questions rated from 0 (best) to 10 (worst). Ankylosing Spondylitis Quality of Life (ASQoL - Spanish validated version) scores from 0 to 18, where lower scores indicate better health-related quality of life. Adverse event (AE) or infection presented as proportion (95% CI) of patients with at least one reported adverse event and/or infection; related presented as proportion (95% CI) of patients with at least one reported AE and/or infection assessed by the investigator as at least possibly related to TNF inhibitor (TNFi) treatment; severe presented as proportion (95% CI) of patients with at least one severe reported AE and/or infection according to standard definitions (fatal or life-threatening, required or prolonged the patient’s hospitalization, caused significant or persistent disability, caused congenital anomaly/birth defect or required immediate medical intervention to avoid any of the previous outcomes); severe and related presented as proportion (95% CI) of patients with at least one reported severe and related AE and/or infection. SER: Spanish Society of Rheumatology (Sociedad Española de Reumatología)

^a^Adjusted percentage estimates [95% CI], binomial regression

^b^Adjusted least square means (standard error of the mean) [95% CI], mixed model for repeated measurements ^c^Number (%) [95CI]), analyzed by Fisher’s exact test

At least one blood sample was available from each of 55 subjects (29 in the full-dose group and 26 in the reduced-dose group) for assessment of plasma levels of inflammatory biomarkers and drugs (Additional file 1: Table S1). All samples tested for TNFi levels were positive, and thus no ADA levels were determined.

Amongst clinical variables at baseline, BASFI, ASQoL and the type of TNFi were all significantly associated with the probability of LDA after 1 year in univariate models, but only the type of TNFi and BASFI remained significant in multivariate models. The treatment effect (dose group assigned) showed no significant heterogeneity according to the BASFI (*p* = 0.5244) or type of TNFi (*p* = 0.887), thus suggesting that the predictive value of BASFI and type of TNFi on the outcome is independent of the assigned dose schedule (full dose or reduced dose) (Additional file 1: Table S2). Considering inflammatory biomarkers and drug plasma levels, only hs-CRP levels were higher in subjects without LDA at 1 year (Additional file 1: Table S3). Univariate models including both clinical and analytical biomarkers had poor predictive value, with the lower CI for the ROC AUC always < 0.62; multivariate methods were unfeasible as no combination of factors improved the univariate models (Additional file 1: Table S3).

Of the 126 randomized patients, serious and related adverse events or infections were reported in 7/62 patients (11.3%) in the full-dose group and 2/61 (3.3%) patients in the reduced-dose group (*p* value = 0.164) (Table 3). A total of 34 infections were reported by 26 subjects, among whom 15 infections were reported by 11/62 subjects (17.7%) in the full-dose group and 4 infections were reported by 3/61 subjects (4.9%) in the reduced-dose group as being at least possibly related to treatment (Additional file 1: Table S4). Treatment was discontinued in 6/62 (9.7%) and 13/61 patients (21.3%) in the full-dose and reduced-dose groups, respectively (*p* value = 0.086), among whom 3/62 (4.8%) in the full-dose and 8/61 (13.1%) in the reduced-dose group had treatment discontinued due to poor disease control, according to physician judgment (*p* value = 0.204).

## Discussion

This trial has shown that a dose-reduction strategy may be non-inferior to full-dose treatment in patients with AS in persistent clinical remission with TNFi, in terms of maintenance of LDA after 1 year. In the reduced-dose group, less serious and related adverse events or infections were observed, but also more withdrawals due to insufficient disease control, although not statistically significant.

Observational studies have reported that complete TNFi discontinuation in patients with AS is not feasible [16] despite 60–80% of patients have LDA at 12–21 months after TNFi dose reduction [19, 26, 27]. However, these studies were uncontrolled or retrospective and sometimes had a small sample size, wide inclusion criteria and many dose schedules, leading to substantial heterogeneity that limited the generalizability of any dose-reduction recommendation.

Our results show that more than 80% and 77% of patients with AS treated with TNFi had LDA or remission at 1 year after randomization to standard or reduced-dose schedules, respectively, in a pragmatic setting, with high external validity. Clear correlation has been reported in patients with AS between BASDAI, BASFI or ASDAS-CRP and disease activity, function and cost-effectiveness of treatments [28–30]. Our study has shown similar results in both randomized arms for these variables. No differences were observed between the groups in the frequency of flares, and no substantial changes in the proportion of NSAID treatments were observed during follow up in either TNFi regimen. However, despite the overall small number of early study withdrawals due to poor disease control, these were numerically higher in the reduced-dose group. This observation has to be interpreted in the context of an open-label study, and considering which the clinical options were in the case of observing worse clinical scores: in the reduced-dose arm, the options included returning to full-dose schedule, while in the full-dose group the options were more aggressive, either dose intensification or switching.

Since there are no universally accepted criteria for remission in AS, we used strict criteria at baseline, and randomized only patients with persistent and maintained clinical remission during the last 6 months while on TNFi [31]. In the absence of an alternative internationally agreed consensus on thresholds for response and control at the time of the study design, we used the Spanish Society of Rheumatology definition for low disease activity [16]. The definition was reflective of a consensus on the clinical therapeutic goal of treatment requiring action if not met. The definition was well-known, widely accepted and applied in clinical practice by rheumatologists in Spain.

The standardized dose-reduction protocol included lengthening the dosing interval by 50% for all subcutaneous TNFi and reducing the infliximab dose from 5 to 3 mg/8 week, according to reported data [15]. This allowed a reduction in the heterogeneity of drug regimes, a limitation reported by other studies [27], although it did not allow evaluation of the potential benefit of additional patient-tailored dose reductions. Most patients were on etanercept and adalimumab, with < 20% of patients on infliximab, consistent with previous reports [12]. Randomization was stratified by drug to ensure comparability of groups; subgroup analysis based on stratification factors suggested that infliximab was associated with a higher risk of failure, although no interaction with the randomized strategy was observed, and thus the risk was similar regardless of whether doses were reduced or not. Considering that patients were not randomized to a given TNFi, but to the dosing strategy, this observation is of limited value for causality assessment, and indication bias cannot be discarded. Similarly, although observational data suggest that dose reduction may be easier with etanercept [14], we found no differences for this stratum.

One goal of personalized medicine is to identify probable beneficiaries of an intervention through clinical or biological markers [18]. We attempted to identify clinical and biological markers of a successful TNFi dose-reduction strategy. Baseline values of BASFI, ASQoL and the TNFi drug were associated with the odds of LDA after 1 year in univariate models; but in multivariate models only TNFi drug and BASFI remained significant. Similarly, hs-CRP was higher in subjects without LDA at 1 year. CRP best reflects objective inflammation and serum CRP is predictive of TNFi response in patients with AS [30].

Although reports have suggested no advantages of hs-CRP as compared with CRP in predicting the clinical response to TNFi [18], the results observed for hs-CRP are not unexpected, since it is a more sensitive technique. Reports have suggested a potential association with TNFi levels in rheumatoid arthritis, but data on AS are controversial [31–33]. We found no association between serum TNFi concentrations and the persistence of LDA at 1 year, although we had only few samples and these were for heterogeneous drugs. All samples had quantifiable TNFi levels, so none of the samples was tested for ADA. Other biomarkers, including calprotectin, did not significantly predict the likelihood of LDA at 1 year in our study. Recently, calprotectin levels have been suggested as a predictor of the clinical response [18], although in acute clinical response, and not in patients in remission.

There were no interactions between dose regimen and TNFi or BASFI, indicating that their predictive value was independent of the actual dose regimen assigned, and multivariate models including biological markers were of low predictive value. Thus, we could not propose any marker to identify patients with higher chances of maintaining a low disease activity after reduction of TNFi dose.

Serious adverse events and infections were nominally less than one third in the reduced-dose group as compared to full dose, although not significant. This suggests low TNFi doses might have a better safety profile, but confirmation is needed, since the size and duration of our study were insufficient to draw conclusions. However, the differences might be relevant, especially since these are long-term treatments for often-fragile patients.

Our study has several limitations. First, due to limited funding and recruitment delays, the final sample size was smaller than anticipated. Although the sample was sufficient to conclude on the pre-determined primary objective, the limited sample size hindered the implementation of secondary objectives. Differences in adverse events were not significant and biological samples were obtained from only half the study population, likely reducing the ability to build a predictive model to identify patients with poorer chances of maintaining LDA after dose-halving. In addition, although the study was designed to include axSpA, only patients with AS were finally included. In addition, the study was not designed to determine which TNFi is more effective, nor powered to determine whether a specific TNFi can be reduced more successfully than another.

A conservative TNFi reduction schedule was applied in this study (about 50% of the recommended dose), and neither the potential benefit of an extra dose reduction or the effects of treatment withdrawal were studied. In clinical practice, infliximab can be reduced more than other TNFi, i.e., dose and interval; however, given the limited statistical power and the fact that this was an unplanned analysis, we cannot venture to show results of subgroup analysis by dose. Likewise, the 12-month follow up was insufficient to determine the potential effects of long-term TNFi reduction, especially on structural damage, which requires 2–4 years to detect relevant changes [31, 34]. A suggestion that TNFi dose tapering may be associated with more rapid radiographic progression than full doses regardless of LDA maintenance [31], especially in patients with baseline syndesmophytes, must be confirmed by prospective studies. Strict monitoring of radiographic progression of patients with AS is recommended if dose reduction is considered [31].

## Conclusion

In spite of a number of limitations, this is the first randomized trial to support the non-inferiority of reduced-dose TNFi doses compared with full doses in patients with SpA. The results were consistent across many variables and TNFi drugs, and suggest a better safety profile with reduced doses, although the safety data must be interpreted with caution due to the small sample size. Overall, the study results support TNFi dose-reduction in patients with AS in stable clinical remission.

## Additional file

Additional file 1: **Table S1.** Baseline characteristics, subset with inflammatory biomarkers. **Table S2.** Baseline predictors of loss of low disease activity at 1 year in the logistic regression analysis. **Table S3.** Univariate methods for loss of low disease activity at 1 year– subset with inflammatory biomarkers. **Table S4.** Listing of infectious adverse events. (DOCX 38 kb)

## Abbreviations

AS: Ankylosing spondylitis
ASAS: Assessment of Spondyloarthritis International Society
ASDAS: Ankylosing Spondylitis Disease Activity Score
ASQoL: Ankylosing Spondylitis Quality of Life
axSpA: Axial spondyloarthritis
BASFI: Bath Anklylosing Spondylitis Functional Index
CI: Confidence interval
CRP: C-reactive protein
DMARD: Disease-modifying anti-rheumatic drug
eCRF: Electronic case report form
ELISA: Enzyme-linked immunosorbent assay
FAS: Full analysis set
hs-CRP: High-sensitivity C-reactive protein
LDA: Low disease activity
NSAID: Non-steroidal anti-inflammatory drug
PPS: Per-protocol set
SD: Standard deviation
SpA: Spondyloarthritis
TNFi: TNF inhibitors
