# Supplementary material for: Non-inferiority of dose reduction versus standard dosing of TNF-inhibitors in axial spondyloarthritis
Source: Arthritis Res Ther. 2019 Jan 8;21:11. doi: 10.1186/s13075-018-1772-z (PMC6323809; doi:10.1186/s13075-018-1772-z)
Supplement: Supplementary file 1 — Table S1. Baseline characteristics, subset with inflammatory biomarkers. Table S2. Baseline predictors of loss of low disease activity at 1 year in the logistic regression analysis. Table S3. Univariate methods for loss of low disease activity at 1 year– subset with inflammatory biomarkers. Table S4. Listing of infectious adverse events. (DOCX 38 kb) [file 13075_2018_1772_MOESM1_ESM.docx]

**Additional file 1: Table S1. Baseline characteristics, subset with inflammatory biomarkers**

|  | Full dose  (N=29) | Reduced dose  (N=26) | Total (n=55) | p-value |
| --- | --- | --- | --- | --- |
| Gender (women) | 26 (89.7%) | 20 (76.9%) | 46 (83.6%) | 0.281 |
| Age | 46.0 (12.7) | 40.8 (12.8) | 43.6 (12.9) | 0.137 |
| BMI | 25.7 (3.4) | 25.4 (3.3) | 25.5 (3.3) | 0.735 |
| Weight | 76.4 (10.8) | 74.6 (13.7) | 75.5 (12.1) | 0.597 |
| TNFi drug |  |  |  | 0.610 |
| Adalimumab | 14 (48.3%) | 15 (57.7%) | 29 (52.7%) |  |
| Etanercept | 10 (34.5%) | 5 (19.2%) | 15 (27.3%) |  |
| Golimumab | 1 (3.4%) | 2 (7.7%) | 3 (5.5%) |  |
| Infliximab | 4 (13.8%) | 4 (15.4%) | 8 (14.5%) |  |
| ASDAS-CRP | 0.9 (0.5) | 0.8 (0.4) | 0.8 (0.5) | 0.561 |
| BASDAI | 1.0 (0.8) | 0.9 (0.7) | 1.0 (0.7) | 0.67 |
| BASFI | 1.2 (1.4) | 0.9 (1.4) | 1.1 (1.4) | 0.458 |
| ASQoL | 2.5 (3.3) | 1.7 (2.0) | 2.1 (2.8) | 0.308 |
| VAS | 0.9 (1.1) | 0.8 (0.8) | 0.9 (0.9) | 0.71 |
| PGA | 1.1 (1.0) | 0.9 (0.9) | 1.0 (0.9) | 0.469 |
| IGA | 0.8 (0.8) | 0.6 (0.7) | 0.7 (0.8) | 0.303 |
| Dug Concentration (standardised units)^a^ | 0.15 (0.92) | -0.17 (1.01) | 0 (0.97) | 0.217 |
| HS-PCR (ng/mL) | 29358 (29822) | 34569 (39476) | 32130 (35201) | 0.298 |
| DKK1 (pg/ml) | 434 (527) | 600 (596) | 512 (561) | 0.278 |
| IL-6 (pg/mL) | 0.1 (0.2) | 1.1 (3.1) | 0.6 (2.2) | 0.144 |
| TNF-a (pg/mL) | 5.1 (6.5) | 3.4 (3.6) | 4.3 (5.4) | 0.254 |
| SOST (pg/mL) | 1916 (1112) | 1563 (813) | 1749 (990) | 0.189 |
| Calprotectin (ng/mL) | 1132 (527) | 1894 (2594) | 1492 (1846) | 0.153 |

Descriptive data are Means (SD) or n (%) as appropriate. BMI: Body Mass Index; TNFi: Tumour Necrosis Factor inhibitor drug; ASDAS-CRP: Ankylosing Spondylitis Disease Activity Score including C Reactive Protein; BASDAI: Bath Ankylosing Spondylitis Disease Activity Index; BASFI: Bath Ankylosing Spondylitis Functional Index; ASQoL: Ankylosing Spondylitis Quality of Life (Spanish validated version); VAS: Nocturnal Axial Pain rated by patient from 0 (none) to 10 (worst). PGA: Patient Global Assessment of disease activity rated from 0 (best) to 10 (worst); IGA: Investigator’s Global Assessment of disease activity rated from 0 (best) to 10 (worst); hs-CRP: high sensitivity C Reactive Protein; DKK1: dickkopf-related protein 1; IL6: interleukin 6; TNFa: Tumour Necrosis Factor alpha, SOST: sclerostin.

a) Analysis conducted with standardised values by type of Drug. Since all the samples were positive for TNFi drug, none of the samples was tested for ADA

**Table S2. Baseline predictors of loss of low disease activity at 1 year in the logistic regression analysis.**

|  | **Low Disease Activity** | | **Univariate** | | **Multivariate ^a^** | |
| --- | --- | --- | --- | --- | --- | --- |
|  | **Yes**  **(n=95)** | **No**  **(n=18)** | **OR**  **[95% CI]** | **P value** | **OR**  **[95% CI]** | **P value** |
| Age | 44.85 (13.13) | 49.39 (12.02) | 1.03 [0.99; 1.07] | 0.178 |  |  |
| Gender (Women) | 80 (84.2%) | 15 (83.3%) | 1.07 [0.27; 4.14] | 0.926 |  |  |
| BMI | 25.78 (3.57) | 26.33 (3.90) | 1.04 [0.91; 1.19] | 0.555 |  |  |
| Weight | 75.53 (12.69) | 74.67 (10.15) | 0.99 [0.95; 1.04] | 0.783 |  |  |
| Years from diagnosis | 12.86 (10.14) | 16.89 (10.48) | 1.04 [0.99; 1.08] | 0.131 |  |  |
| Drug |  |  |  | **0.005** |  | 0.009 |
| Adalimumab | 39 (41.1%) | 5 (27.8%) | 1 (Ref.) |  | 1 (Ref.) |  |
| Etanercept | 35 (36.8%) | 3 (16.7%) | 0.67 [0.15; 3.00] |  | 0.41 [ 0.08; 2.19] |  |
| Golimumab | 9 (9.5%) | 0 (0.0%) | NA |  | NA |  |
| Infliximab | 12 (12.6%) | 10 (55.6%) | 6.50 [1.86; 22.76] |  | 5.24 [ 1.42; 19.38] |  |
| Treatment (reduced dose) | 48 (50.5%) | 10 (55.6%) | 1.22 [0.44; 3.37] | 0.696 |  |  |
| Previous TNFi | 21 (22.1%) | 4 (22.2%) | 0.99 [0.30; 3.34] | 0.991 |  |  |
| Previous dose reduction | 2 (2.1%) | 1 (5.6%) | 0.37 [0.03; 4.26] | 0.422 |  |  |
| ASDAS-CRP | 0.74 (0.37) | 0.83 (0.46) | 1.85 [0.49; 6.90] | 0.362 |  |  |
| BASDAI | 0.96 (0.67) | 1.09 (0.81) | 1.32 [0.64; 2.72] | 0.460 |  |  |
| BASFI | 1.36 (1.63) | 2.88 (2.63) | 1.41 [1.11; 1.78] | **0.005** | 1.47 [ 1.11; 1.95] | 0.007 |
| ASQUOL | 2.04 (2.56) | 3.56 (3.28) | 1.19 [1.01; 1.40] | **0.039** |  |  |
| VAS | 0.85 (1.00) | 1.33 (1.41) | 1.44 [0.94; 2.21] | **0.091** |  |  |
| PGA | 1.31 (1.47) | 1.67 (1.41) | 1.16 [0.85; 1.59] | 0.341 |  |  |
| IGA | 0.91 (0.86) | 0.89 (0.76) | 0.98 [0.54; 1.78] | 0.940 |  |  |
| CRP (mg/L) | 0.84 (0.58) | 1.09 (0.63) | 1.98 [0.85; 4.58] | 0.112 |  |  |

Descriptive data are Means (SD) or n (%) as appropriate. 95%CI: 95% confidence interval for estimator. Ref: reference category. BMI: Body Mass Index; TNFi: Tumour Necrosis Factor inhibitor drug; ASDAS-CRP: Ankylosing Spondylitis Disease Activity Score including C Reactive Protein; BASDAI: Bath Ankylosing Spondylitis Disease Activity Index; BASFI: Bath Ankylosing Spondylitis Functional Index; ASQoL: Ankylosing Spondylitis Quality of Life (Spanish validated version); VAS: Nocturnal Axial Pain rated by patient from 0 (none) to 10 (worst). PGA: Patient Global Assessment of disease activity rated from 0 (best) to 10 (worst); IGA: Investigator’s Global Assessment of disease activity rated from 0 (best) to 10 (worst); CRP: C Reactive Protein (routine measurement)

a) Stepwise multivariate logistic regression including all variables with p<0.1 at the univariate testing. Only BASFI and the stratum drug were included in the final multivariate model, ROC AUC [95%CI: 0.8481 [0.7549-0.9413]. No significant interaction was observed with the treatment effect (full vs reduced dose) for either BASFI (p = 0.5244) nor TNFi stratum (p=0.8868).

**Table S3. Univariate methods for loss of low disease activity at 1 year– subset with inflammatory biomarkers**

|  | **Low Disease Activity** | | **Univariate^c^** | |
| --- | --- | --- | --- | --- |
|  | **Yes**  **(n=49)** | **No**  **(n=6)** | **OR**  **[95% CI]** | **p-value** |
| Gender | 8 (16.3%) | 1 (16.7%) | 1.03 [0.11; 9.99] | 0.983 |
| Age | 43.16 (12.45) | 47.00 (16.80) | 1.02 [0.96; 1.09] | 0.490 |
| BMI | 25.32 (3.33) | 26.60 (3.30) | 1.12 [0.87; 1.45] | 0.372 |
| Weight | 75.22 (12.52) | 78.05 (8.75) | 1.02 [0.95; 1.09] | 0.588 |
|  |  |  | 1.01 [0.92; 1.10] | 0.896 |
| Treat (Dose reduction) | 24 (49.0%) | 2 (33.3%) | 0.52 [0.09; 3.11] | 0.474 |
| Previous TNFi (no) | 9 (18.4%) | 1 (16.7%) | 1.12 [0.12; 10.84] | 0.919 |
| TNFi Drug |  |  |  | 0.391 |
| Adalimumab | 26 (53.1%) | 3 (50.0%) | 1 (Ref.) |  |
| Etanercept | 15 (30.6%) | 0 (0.0%) | NA |  |
| Golimumab | 3 (6.1%) | 0 (0.0%) | NA |  |
| Infliximab | 5 (10.2%) | 3 (50.0%) | 5.20 [0.81; 33.56] |  |
| ASDAS-CRP | 0.66 (0.32) | 0.74 (0.46) | 2.13 [0.17; 26.92] | 0.559 |
| BASDAI | 0.88 (0.62) | 1.03 (0.64) | 1.49 [0.38; 5.89] | 0.569 |
| BASFI | 1.04 (1.09) | 2.33 (1.51) | 2.18 [1.10; 4.33] | **0.026** |
| ASQUOL | 1.58 (2.22) | 3.17 (2.23) | 1.28 [0.93; 1.77] | 0.125 |
| VAS | 0.67 (0.85) | 0.83 (0.98) | 1.23 [0.48; 3.20] | 0.665 |
| PGA | 0.96 (0.93) | 1.00 (1.10) | 1.05 [0.43; 2.58] | 0.920 |
| IGA | 0.80 (0.71) | 0.83 (0.75) | 1.08 [0.32; 3.61] | 0.901 |
| Dug Concentration (standardised units) ^b^ | -0.09 (0.86) | 0.77 (1.52) | 2.3 [0.98 – 5.37]] | 0.055 |
| HS-CRP ^a^ | 28874 (31865) | 71390 (58507) | 217.12 [2.19; 21574.69] | **0.022** |
| DKK1^a^ | 535.97 (587.7) | 317.46 (192.8) | 0.47 [0.08; 2.74] | 0.400 |
| IL6 | 0.24 (0.58) | 3.31 (6.16) | 1.95 [0.73 -5.21] | 0.094 |
| TNFa | 4.57 (5.60) | 1.98 (0.86) | 0.66 [0.33 -1.33] | 0.279 |
| SOST^a^ | 1684 (898) | 2278 (1568) | 1.64 [0.80; 3.36] | 0.176 |
| Calprotectin^a^ | 1535 (1946) | 1144 (502) | 0.67 [0.13; 3.56] | 0.637 |

Descriptive data are Means (SD) or n (%) as appropriate. BMI: Body Mass Index; TNFi: Tumour Necrosis Factor inhibitor drug; ASDAS-CRP: Ankylosing Spondylitis Disease Activity Score including C Reactive Protein; BASDAI: Bath Ankylosing Spondylitis Disease Activity Index; BASFI: Bath Ankylosing Spondylitis Functional Index; ASQoL: Ankylosing Spondylitis Quality of Life (Spanish validated version); VAS: Nocturnal Axial Pain rated by patient from 0 (none) to 10 (worst). PGA: Patient Global Assessment of disease activity rated from 0 (best) to 10 (worst); IGA: Investigator’s Global Assessment of disease activity rated from 0 (best) to 10 (worst); hs-CRP: high sensitivity C Reactive Protein; DKK1: dickkopf-related protein 1; IL6: interleukin 6; TNFa: Tumour Necrosis Factor alpha, SOST: sclerostin.

a) OR and 95%CI are given per 1 SD increase for these marked variables.

b) all analyses conducted with standardised values by type of Drug

c) No valid multivariate model was identified. The ROC AUC for the significant univariate models at the two-sided 10% alpha level were: IL6: 0.718 [0.497-0.939]; BASFI: 0.774 [0.611- 0.937], HS-CRP: 0.714 [0.411-1.00], Drug concentration level: 0.632 [0.364 – 0.901]

**Table S4. Listing of infectious adverse events**

| **Adverse events reported:**  **MedDRA Preferred term** | **Full dose (N=62)**  **N (N Related)** | **Reduced dose (N=61)**  **N (N Related)** | **Total (N=123)**  **N (N related)** |
| --- | --- | --- | --- |
| Dental infection |  | 1 (0) | 1 (0) |
| Ear infection |  | 2 (0) | 2 (0) |
| Flu infection / Flu syndrome | 2 (0) | 1 (0) | 3 (0) |
| Gastrointestinal infections | 1 (0) | 2 (1) | 3 (1) |
| Lower respiratory tract and lung infections / Bronchitis | 2 (0) | 2 (1) | 4 (1) |
| Skin infection | 2 (2) |  | 2 (2) |
| Upper respiratory tract infection | 10 (7) | 6 (2) | 16 (9) |
| Varicella / Herpes zoster | 2 (1) | 1 (0) | 3 (1) |
| Total number of reported events | 19 (10) | 15 (4) | 34 (14) |
| Total number of patients involved | 15 (7) | 11 (3) | 26 (10) |

Related adverse events: Events with causality assessment by investigator reported as Possible, Probable or Certain. Not related: Events with causality assessment by investigator reported as Not related or Not assessable.
